# Obesity-Related Changes in Human Plasma Lipidome Determined by the Lipidyzer Platform

**DOI:** 10.3390/biom11020326

**Published:** 2021-02-21

**Authors:** Péter Pikó, László Pál, Sándor Szűcs, Zsigmond Kósa, János Sándor, Róza Ádány

**Affiliations:** 1 MTA-DE Public Health Research Group, University of Debrecen, 4032 Debrecen, Hungary; piko.peter@med.unideb.hu; 2 Department of Public Health and Epidemiology, Faculty of Medicine, University of Debrecen, 4032 Debrecen, Hungary; pal.laszlo@med.unideb.hu (L.P.); szucs.sandor@med.unideb.hu (S.S.); sandor.janos@med.unideb.hu (J.S.); 3 Department of Health Methodology and Public Health, Faculty of Health, University of Debrecen, 4400 Nyíregyháza, Hungary; kosa.zsigmond@foh.unideb.hu

**Keywords:** obesity, body mass index (BMI), lipidomic analysis, Lipidyzer platform, exploratory principal component analysis, stepwise regression analysis, lipid species ratio

## Abstract

Obesity is an increasing public health concern both in the developed and developing countries. Previous studies have demonstrated that considerable alterations in lipid metabolism and consequently marked changes in lipid profile are associated with the onset and progression of obesity-related complications. To characterize the full spectrum of obesity-induced changes in lipid metabolism, direct infusion tandem mass spectrometry analysis is the most promising approach. To better understand which of the many lipid species are the most strongly associated with obesity, the aim of our work was to measure and profile plasma lipids in normal (*n* = 57), overweight (*n* = 31), and obese (*n* = 48) individuals randomly selected from samples of Hungarian general and Roma populations by using the targeted quantitative lipidomics platform, the Lipidyzer. Principal component and stepwise regression analyses were used to identify the most significant clusters and species of lipids by increasing body mass index (BMI). From the 18 clusters identified four key lipid species (PE P-16:0/20:3, TG 20:4_33:1, TG 22:6_36:4, TG 18:3_33:0) showed a strong significant positive and three others (Hex-Cer 18:1;O2/22:0, LPC 18:2, PC 18:1_18:1) significant negative association with BMI. Compared to individual lipid species alone, the lipid species ratio (LSR) we introduced showed an extremely strong, at least 9 orders of magnitude stronger, association with BMI. The LSR can be used as a sensitive and predictive indicator to monitor obesity-related alterations in human plasma and control the effectiveness of treatment of obesity associated non-communicable diseases.

## 1. Introduction

Obesity is an increasing public health concern both in the developed and developing countries [1,2]. Globally, its prevalence increased significantly between 1975 and 2016 [2]. The latest available data from the World Health Organization (WHO), from 2016, showed that 1.9 billion adults were overweight of which 650 million obese worldwide [2]. It can play a pivotal role in the development of several chronic pathological conditions and diseases including insulin resistance, type-2 diabetes, cardiovascular diseases, musculoskeletal disorders, fatty liver disease, and cancer [1,2]. As a result, more than 5 million deaths and 160 million disability adjusted life years were attributable to obesity-related diseases in 2019 [3]. Previous studies have demonstrated that obesity is not simply an increase in the mass of body adipose tissue, but it is associated with considerable alterations in different metabolic pathways, among them in lipid homeostasis [4]. The altered concentrations of plasma lipids such as cholesterol, triacylglycerols, and low- and high-density lipoproteins in the above-mentioned noncommunicable diseases (NCDs) have been well known for a long time, and changes in the level of these lipid variants are widely monitored in routine clinical practice to detect the development and progression of NCDs [5,6]. Although these regularly determined laboratory parameters provide information on lipid disturbances in general, today it is widely accepted that characterization of the full spectrum of obesity-induced changes in lipid metabolism, i.e., a detailed analysis of the human plasma lipidome, is required to create attractive hypotheses on the pathomechanisms of obesity-related NCDs and identify sensitive predictive and prognostic biomarkers, as well as targets to their prevention and therapy [7].

Recently, high-resolution lipidomic methods using high-performance liquid chromatography (HPLC) combined with tandem mass spectrometry (MS-MS) have enabled a comprehensive analysis of human plasma lipidome by simultaneous determination of several hundreds of lipid species covering the main lipid families including fatty acids, glycerolipids, glycerophospholipids, sphingolipids, sterol lipids, and prenol lipids [7,8,9]. These techniques also allowed the identification of a wide range of specific lipid molecules that can reflect disturbances in lipid metabolism in obesity [9]. Although a large number of lipid species showing an association with obesity were detected previously, findings are quite diverse both from qualitative and quantitative approach [10,11,12,13]. In one study, concentrations of triacylglycerol species (TGs) including TG 56:4, TG 56:5, and lysophosphatidylcholines (LPCs) LPC 18:0 and LPC 16:0 (for detailed explanation of the nomenclature of lipid species see the *Material and Methods* section) were positively correlated with body mass index (BMI) [10]. In another investigation, LPC 18:1 and LPC 18:2 was found to be negatively related to waist circumference [11]. In addition to these LPC species, the levels of LPC 20:0, LPC 20:1, and LPC 20:2 were also significantly lower in the obese group compared with normal weight individuals [12]. As reported by Rauschert et al., the concentrations of phosphatidylcholines (PCs) such as PC 38:3, PC 38:4, PC 38:5, PC 40:5, PC 40:6, and sphingomyelin species (SMs), namely SM 32:2;O2, SM 33:2;O2, SM 34:2;O2, SM 34:3;O2, SM 36:0;O2, SM 36:2;O2, SM 36:3;O2, SM 40:2;O2, SM 42:3;O2, SM 42:4;O2, and SM 43:3;O2, were positively associated with waist circumference [11]. Most recently, among the 154 circulating lipid species investigated on selected samples from the offspring and third generations of the Framingham Heart Study, Yin et al. have found 39 obesity-associated lipid species belonging to lipid classes of diacylglycerols (DGs), LPCs, lysophosphatidyethanolamines (LPEs), PCs, SMs, and TGs [13]. In this study, the authors reported that the levels of LPCs, PCs, and LPEs were inversely associated, while SMs, TGs, and DGs directly associated with obesity [13].

Due to the diverse findings of previous studies carried out in different study populations with various lipidomic methods targeting the analysis of heterogeneous types of lipids, using different informatics and computational strategies with no unified algorithm for data handling to analyze the large mass of data obtained, the interpretation of obesity-related alterations in lipid metabolism is very difficult [14,15].

To further examine which of the many lipid species are, and how they are, associated with obesity, our work had three objectives. First, to profile plasma lipids in normal, overweight, and obese individuals by using the Lipidyzer platform, which is suitable for simultaneous determination of 1100 lipid species covering 13 lipid classes and thereby eliminating possible bias including pre-analytical ones arising from methodological heterogeneity [16]. Second, to apply a hierarchical statistical procedure for selection of lipidomic data relevant for human pathology. Third, using this statistical approach, to identify the most significant clusters of lipid molecules in obesity and from them select the species strongly associated with BMI.

The results presented in this study showed that four and three key lipid species demonstrated a strong significant positive (PE P-16:0/20:3, TG 20:4_33:1, TG 22:6_36:4, TG 18:3_33:0) and negative (Hex-Cer 18:1;O2/22:0, LPC 18:2, PC 18:1_18:1) association with BMI, respectively.

## 2. Materials and Methods

### 2.1. Study Design and Population

**Figure 1:**
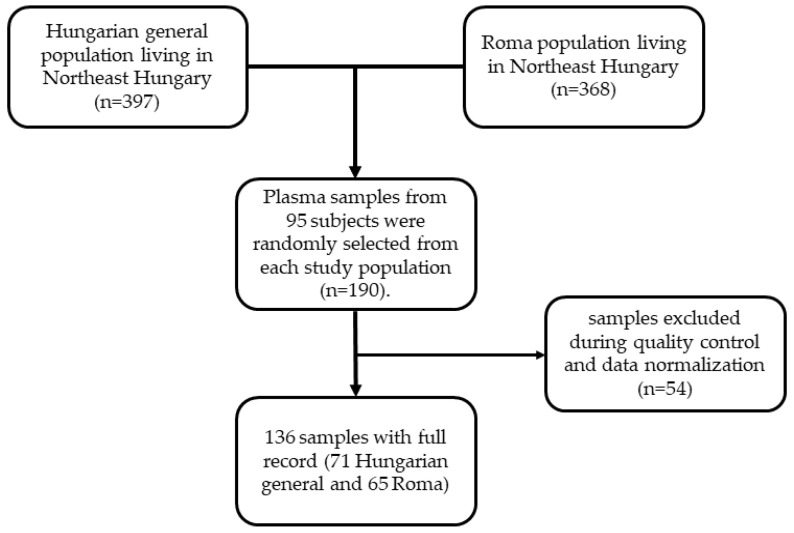
Flowchart showing the selection of study population and plasma samples for lipidomic analysis.

Study design and data collection were described in detail in our previous study [17]. In brief, a complex health survey was designed to form a complex database for comparative and association studies to better understand the background of the very unfavorable health of Roma individuals in comparison with the general population, especially the high burden of cardiometabolic diseases. This cross-sectional study had three main sources of data including questionnaire-based, physical, and laboratory examinations involving adults aged 20–64 years from the Hungarian general (HG) and Hungarian Roma (HR) populations. Altogether 832 participants were recruited in the study including 417 HG (185 men and 232 women) and 415 HR (108 men and 307 women) subjects. From them, 35 (7 HG and 28 HR) and 32 (13 HG and 19 HR) participants were excluded due to missing anthropometric and/or laboratory parameters. In addition to anthropometric, demographic, socioeconomic, and health-related data, fasting blood samples (native and EDTA-anticoagulated) were also collected for routine laboratory tests, genetic, and lipidomic investigations. Regarding lipidomic analysis, plasma samples were separated by centrifugation and kept at −80 °C until lipid extraction. From the remaining 765 (397 HG and 368 HR) persons, 190 (95 HG and 95 HR) were randomly selected for this study. The flowchart showing the process of selection of study population and plasma samples is presented in Figure 1. The participants were categorized as normal weight, overweight, and obese subjects with BMIs of less than 25.0 kg/m^2^, between 25.0 and 29.9 kg/m^2^, and equal or larger than 30.0 kg/m^2^, respectively.

### 2.2. Materials

Methanol, 2-propanol, dichloromethane, water, ammonium-acetate were purchased from VWR International, LLC (Radnor, Radnor, PA, USA). All of them were of HPLC grade. Internal standard (ISTD) kits for quantitative lipidomic analysis of human samples were bought from AB Sciex Germany GmbH (Darmstadt, Germany). The kits contain ISTDs for 13 lipid classes including ceramides (Cers), cholesterolesters (CEs), DGs, acylceradmides (ACer), fatty acids (FAs), hexosylceramides (HexCers), lactosylceramides (LacCers), LPCs, LPEs, PCs, phosphatidylethanolamines (PEs), SMs, and TGs. The composition of ISTD standard mixtures containing isotope-labelled lipid molecules were described in detail previously [18]. Spike standards with quality control plasma kits, SelexION tuning kits, and system suitability test kits were also obtained from AB Sciex Germany GmbH.

### 2.3. Extraction of Lipids

Lipids were extracted from human plasma samples using a modified Bligh-Dyer method [16,18,19,20]. Briefly, plasma samples of 100 µL were mixed with 900 µL of HPLC grade water, 2000 µL of methanol, and 900 µL of dichloromethane in glass centrifuge tubes and vortexed for 5 s. To prepare a quality control sample, 100 µL of quality control plasma (QC) was mixed with 900 µL of HPLC grade water, 2000 µL of methanol, and 900 µL of dichloromethane in another glass centrifuge tube and vortexed for 5 s. To make a spiked sample, 50 µL of quality control spike standard (QC spike) was mixed with 100 µL of QC, 900 µL of HPLC grade water, 2000 µL of methanol, and 900 µL of dichloromethane in a separate glass centrifuge tube and vortexed for 5 s. To compensate for background FA contamination, a blank sample was also prepared by mixing 1000 µL of HPLC grade water, 2000 µL of methanol, and 900 µL of dichloromethane in a glass centrifuge tube and vortexed for 5 s. Then, 100 µL of ISTD mixture was added to each sample (plasma samples, quality control sample, spiked sample, blank sample), vortexed for 5 s, and incubated at room temperature for 30 min. Following incubation, 1000 µL of HPLC grade water and 900 µL of dichloromethane were added to the samples. The samples were vortexed for 5 s and centrifuged at 1000 g at 20 °C for 10 min. Following centrifugation, the samples were separated into an organic (lower phase) and an aqueous phase (upper phase). The organic phases containing the lipid extracts were transferred into separate glass tubes. To obtain the remaining lipids, the extraction was repeated by adding 1800 of µL dichloromethane to the aqueous phase of each sample. Then, the samples were vortexed for 5 s and centrifuged at 1000 g at 20 °C for 10 min to separate them into an organic and an aqueous phase again. The organic phases were collected again and combined with the previous extracts. Subsequently, the extracts were dried completely under nitrogen flow and reconstituted in 250 µL 1:1 mixture of dichloromethane and methanol solution containing 10 mM ammonium-acetate as described in the standard protocol developed by experts of Sciex [16]. Finally, they were transferred into vials for lipidomic analysis.

### 2.4. Lipidomic Analysis

Analyses of lipid samples were carried out using a HPLC coupled with electrospray ionization tandem mass spectrometry (HPLC ESI-MS-MS) as described previously [18]. The Lipidyzer platform consisting of a Nexera X2 HPLC (Shimadzu Germany GmbH, Duisburg, Germany) and a Sciex QTRAP 5500 system equipped with SelexION technology (AB Sciex Germany GmbH, Darmstadt, Germany) was used for lipidomic analysis. A set of 750 × 0.05 mm and 350 × 0.05 mm nanoViper capillary tubes (Thermo Fisher Scientific Inc., Waltham, MA, USA) were used to connect HPLC autosampler valve to the grounding union of the ESI ionization source and the grounding union to the ESI electrode (65 µm internal diameter), respectively. The introduction of 50.0 µL of extracted lipid samples was carried out by flow injection at a flow rate of 7.0 µL/minute. The sample running solution was 1:1 mixture of dichloromethane and methanol containing 10 mM ammonium-acetate. Sample carry-over was minimalized by using zero dead volume nanoViper capillary tubes and washing the capillaries with a 1:1 mixture of dichloromethane and methanol at a flow rate of 30.0 µL/minute for 2 min after each injection. Each sample was measured twice, first with SelexION differential mobility spectrometric separation (DMSS) and then without it. The whole running time was 21 min including time periods of washing of the injector before each run, injection and pumping of 50 µL of sample to the electrode, measurement in positive/negative ion mode, and washing of the capillary tubing after each run. The principle of DMSS is that each lipid class has a specific head group dipole moment that results in differences in the mobility of ions derived from different lipid molecules when a specific compensation voltage (COV) is applied. Sequential analysis of lipid classes can be achieved by changing the COV in the differential mobility unit of the Lipidyzer platform. To improve DMSS, 1-propanol, as a chemical modifier, was added to the curtain gas. Lipidomic analysis were carried out with the following DMSS settings: temperature: low, separation voltage: 3.5 kV, and differential mobility spectrometric resolution: low. Multiple reaction monitoring and switching between positive and negative ionization was used to detect and quantify lipid species. Negative ionization was used with DMSS for the measurement of PCs, PEs, and LPCs and without DMSS for the determination of FAs. Positive ionization was applied with DMSS for the analysis of SMs and without DMSS for the measurement of TGs, DGs, CEs, and Cers. The following mass spectrometer (MS) settings were used: curtain gas: 17, collisionally activated dissociation gas: medium, ion spray voltage: 4.1 kV in positive ionization mode and −2.5 kV in negative ionization mode, temperature: 200 °C, nebulizing gas: 17, and heater gas: 25. QC and QC spike samples were included in each batch containing 8 plasma samples. System control, data acquisition, and analysis were performed automatically with the Lipid Workflow Manager software (AB Sciex Germany GmbH, Darmstadt, Germany). The concentrations of lipid species were obtained in nmol/gram plasma automatically. To ensure data quality, the differential mobility unit, orifice plate, and QJet Ion Guide were manually cleaned with 1:1 mixture of dichloromethane and methanol every week. Following cleaning, the differential mobility unit was tuned with a SelexION kit and a system suitability test was run.

### 2.5. Nomenclature of Lipids

The nomenclature of lipids proposed by the Lipid Maps Consortium was used in this study [21]. TG species containing three fatty acid chains were presented as the number of carbon atoms and double bonds in one of fatty acid chain and the sum of the number of carbon atoms and double bonds in the remaining two fatty acid chains for example TG 20:4_33:1. The other lipid species containing one or two fatty acid chains were demonstrated as the number of carbon atoms and double bonds in each fatty acid chain for example CE 14:0 (contains one fatty acid chain with no double bond), and PC 18:1_18:1 (contains two fatty acid chains both with one double bond).

### 2.6. Statistical Analysis

**Figure 2:**
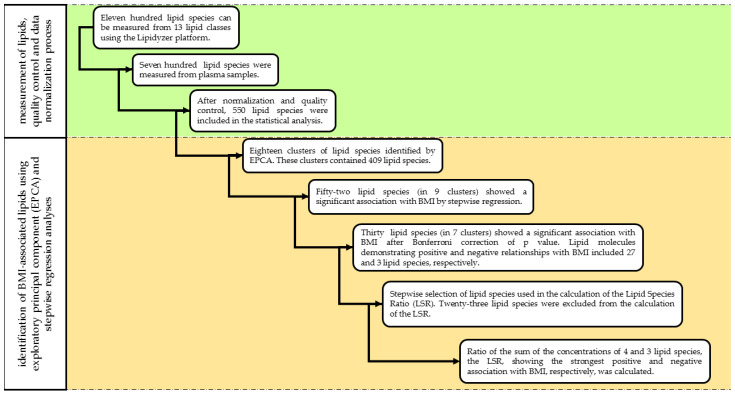
Flowchart of statistical analysis.

All statistical analyses were performed with the IBM SPSS software (version 26, IBM Company, Armonk, NY, USA). For better comparison with the results of previous studies (Bowden et al., 2017; Sales et al., 2016), the lipid concentrations obtained in nmol/gram plasma were transformed to µmol/L assuming that 1 g of plasma is equal to 1 mL of plasma as described by Morigny et al. [22]. The concentrations of each lipid species including FAs were adjusted by blank correction. If the concentrations of lipid species in the QC sample in a batch were outside ± 20 % of their respective nominal values, the whole batch was excluded from the analysis [23]. Therefore, data on plasma samples from 54 subjects were excluded from the statistical analysis. Following normalization, data on the remaining 136 plasma samples were further analyzed. The normality of data on the age, sex, concentration of lipid classes, and species was tested by the Shapiro–Wilk test. When it was found to be non-normally distributed, the Templeton’s two-step approach was used to normalize data [24]. Differences in concentrations and representations of lipid classes in plasma samples from normal weight, overweight, and obese subjects were determined by Mann–Whitney U tests. The Jonckheere-Terpstra test was used to compare mean values across the ordered BMI categories (normal weight, overweight, obese) and clinical parameters of the study population, and concentration of lipid classes [25]. Values of *p* < 0.05 were considered to be statistically significant. The flowchart showing each step of statistical analysis is illustrated in Figure 2.

#### 2.6.1. Factor Analysis

To identify the clusters of lipid species showing an association with BMI, an exploratory principal component analysis (EPCA) was performed including 550 lipid molecules. This method was used to form clusters of lipid species characterizing variance in lipidomic data. Clusters of individual lipid molecules (factors) with eigenvalues of less than 2.0 were excluded from the analysis. The association of the identified clusters with BMI was tested by multivariable linear regression analyses adjusted by sex, age, and ethnicity. Varimax (orthogonal) rotation was applied to identify the lipid species in those clusters that demonstrate an association with BMI. Values of *p* < 0.05 were considered as statistically significant.

#### 2.6.2. Stepwise Regression Analysis

From the clusters, lipid species with absolute loadings greater than 0.5 and belonging to one cluster only were further investigated. To identify the lipid species related to BMI within the clusters, a stepwise regression analysis was carried out separately for each cluster. All analyses were also adjusted for sex, age, and ethnicity. Lipid species showing an association with BMI with p values of less than 0.05 were selected for further analysis.

#### 2.6.3. Calculation of the Lipid Species Ratio

To form a parameter showing a stronger association with BMI than individual lipid molecules alone, the lipid species ratio (LSR) was calculated. Following stepwise regression, those lipid species that demonstrated a significant relationship with BMI were selected for further LSR analysis. To calculate a threshold of statistically significant p values for multiple stepwise regressions, the Bonferroni correction was applied (conventional *p* value of 0.05 divided by the number of independent analyses) and used in the calculation of LSR. Subsequently, the selected lipid molecules were ranked according to the strength of their association, indicated by negative log_10_-transformed p value, with BMI. Next, the concentration of the lipid molecule showing the strongest positive association with BMI was added to the level of the lipid species demonstrating the second strongest positive relationship with BMI. This sum was used for a new regression analysis to test its strength of association with BMI. An increase in the p value indicated a weaker association with BMI than individual lipid molecules alone, while a decrease in the *p* value showed an increase in the strength of relationship with BMI. This procedure was repeated with all of the lipid species showing a positive association with BMI. Subsequently, only the concentrations of those lipid molecules were summed that increased the association with BMI. The same procedure was repeated with the lipid molecules showing a negative association with BMI. Finally, the ratio of the sum of the concentrations of the selected lipid species showing a positive and negative association with BMI (LSR), respectively, was calculated. The concentration of lipid species showing positive and negative association with BMI were included in the numerator and denominator of LSR, respectively (see Equation (1)). The strengths of association between LSR and BMI was tested by a regression analysis.

Equation (1)

(1) $$\mathrm{LSR}=\frac{\mathrm{sum}\;\mathrm{of}\;\mathrm{concentrations}\;\mathrm{of}\;\mathrm{lipid}\;\mathrm{molecules}\;\mathrm{showing}\;\mathrm{positive}\;\mathrm{association}\;\mathrm{with}\;\mathrm{BMI}}{\mathrm{sum}\;\mathrm{of}\;\mathrm{concentrations}\;\mathrm{of}\;\mathrm{lipid}\;\mathrm{molecules}\;\mathrm{showing}\;\mathrm{negative}\;\mathrm{association}\;\mathrm{with}\;\mathrm{BMI}}$$

#### 2.6.4. Estimation of Reference Values for the Sum of the Concentrations of Lipid Species Showing Positive and Negative Association with Body Mass Index and Lipid Species Ratio

To distinguish normal weight and obese subjects, reference values were determined by calculating cut-off points for the sum of the concentrations of lipid species showing positive and negative association with BMI as well as LSR. To find an optimal cut-off value, the receiver operating characteristic curve (ROC) was used. To optimize the sensitivity and specificity of each value, the Youden’s J statistic was applied [26]. The Youden’s indices were calculated for all points of the ROC curves. To determine the optimal cut-off values, the maximum of Youden’s indices were selected for the sum of the concentrations of lipid species showing positive and negative association with BMI as well as LSR.

## 3. Results

### 3.1. Characteristics of Study Population

One hundred and thirty-six individuals were included in this study. The mean age of the participants was 45.02 ± 11.03 years. The demographic and anthropometric parameters as well as laboratory parameters of the study population stratified as normal-, overweight, and obese subjects are demonstrated in Table 1 and Table 2, respectively. The levels of total cholesterol (TC: *p* < 0.01), TG (*p* < 0.001), low-density lipoprotein-cholesterol (LDL-C: *p* < 0.05), and apolipoprotein B (apoB) (*p* < 0.001) were significantly increased while the concentration of HDL-cholesterol significantly decreased (*p* < 0.05) as the BMI categories increased. A statistically significant increasing trend was also observed in case of the TG/HDL ratio throughout the BMI categories (*p* < 0.01).

### 3.2. Associations between Lipid Classes and Body Mass Index

**Figure 3:**
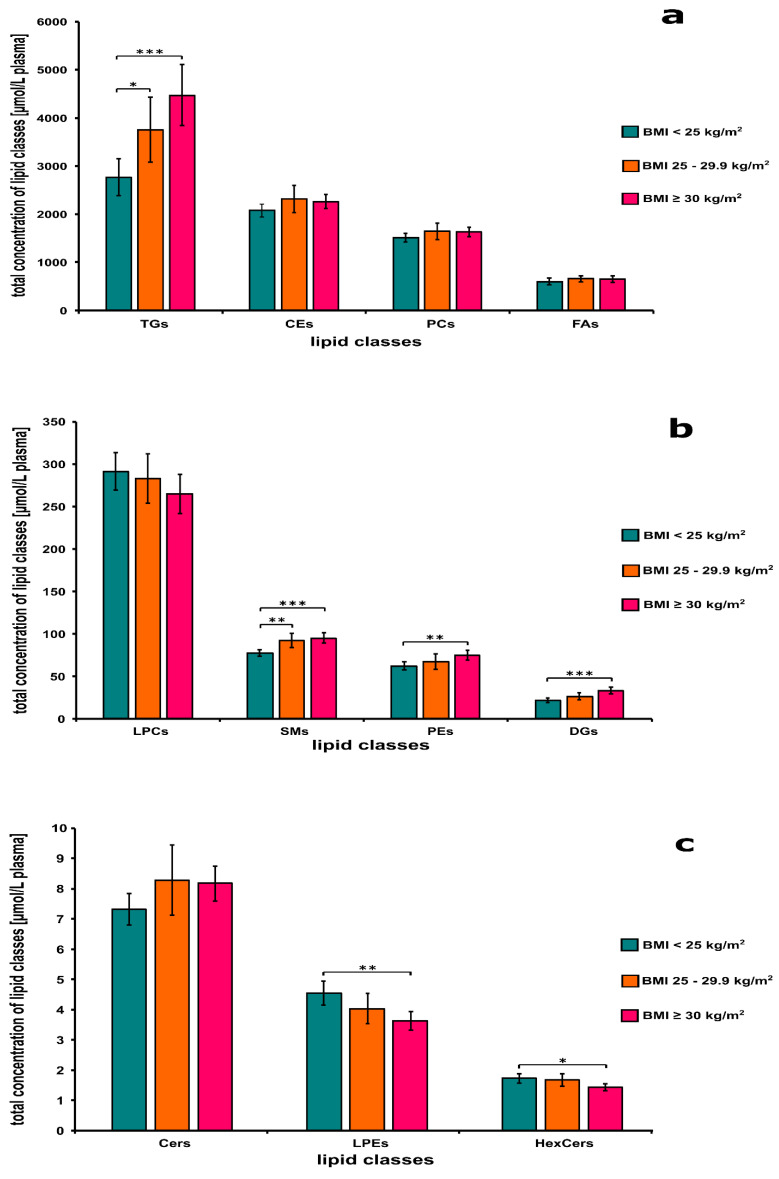
Changes in the average concentration (panels **a**–**c**) of lipid classes in association with body mass index (BMI) categories. Lipids were extracted from plasma samples of normal weight (BMI < 25 kg/m^2^), overweight (BMI: 25–29.9 kg/m^2^), and obese (BMI ≥ 30 kg/m^2^) subjects. The levels of lipid classes were measured by a high-performance liquid chromatography coupled with electrospray ionization tandem mass spectrometry (HPLC ESI-MS-MS) using the Lipidyzer platform as described in the Material and Methods section. Abbreviations: CEs = cholesterolesters, Cers = ceramides, DGs = diacylglycerols, FAs = fatty acids, HexCers = hexosylceramides, LPCs = lysophosphatidylcholines, LPEs = lysophosphatidylethanolamines, PCs = phosphatidylcholines, Pes = phosphatidylethanolamines, SMs = sphingomyelins, TGs = triacylglycerols. * *p* < 0.05; ** *p* < 0.01; *** *p* < 0.001.

Figure 3a shows the concentrations of TGs, CEs, PCs, and FAs in the plasma from normal weight, overweight, and obese individuals. There was a significant difference between the concentration of TGs in the plasma from normal weight and obese (*p* < 0.001) as well as normal weight and overweight persons (*p* < 0.05), respectively (Figure 3a). The levels of TGs and CEs showed significantly increasing trends with BMI categories (*p* < 0.001 for both classes).

Figure 3b presents the levels of LPCs, SMs, PEs, and DGs classes in the plasma from normal weight, overweight, and obese individuals. There was a significant difference between the concentration of SMs in the plasma from normal weight and overweight (*p* < 0.01) as well as normal weight and obese persons (*p* < 0.001), respectively (Figure 3b). Significant difference between the concentrations of PEs (*p* < 0.01) and DGs (*p* < 0.001) in the plasma was observed also from normal weight and obese persons. The levels of SMs (*p* < 0.001), PEs (*p* < 0.01), and DGs (*p* < 0.001) showed significantly increasing trends with BMI categories.

Figure 3c demonstrates the concentrations of Cers, HexCers, and LPEs in the plasma from normal weight, overweight, and obese individuals. There was a significant difference between the levels of LPEs (*p* < 0.01) and HexCers (*p* < 0.05) in the plasma from normal weight and obese individuals (Figure 3c). The concentration of Cers (*p* < 0.05) showed a significantly increasing, while the concentrations of LPEs (*p* < 0.001) and HexCers (*p* < 0.05) demonstrated a significantly decreasing trend with BMI categories.

### 3.3. Results of Factor Analysis

Five hundred and fifty lipid species comprising 5 Cer, 14 CE, 12 DG, 8 FA, 2 HexCer, 9 LPC, 4 LPE, 40 PC, 28 PE, 4 SM, and 424 TG were included in the factor analysis. Eighteen clusters of individual lipid molecules (factors) explaining 92.43% of the total variance with eigenvalues of ≥2 were identified. From the 18, 7 clusters showed significant association with BMI explaining 73.49% of total variance. Their association was independent of the sex, age, and ethnicity of the participants. Of the seven clusters of lipid molecules, four demonstrated positive (cluster 1, 2, 5, and 6) and three negative (cluster 8, 9, and 16) association with BMI. Clusters showing positive relationships with BMI included 307 lipid species belonging to the classes of CEs, DGs, PCs, PEs, and TGs and clusters presenting negative association with BMI comprised 12 lipid species from PCs, PEs, LPCs, and LPEs lipid classes.

From the 18 clusters, lipid species with absolute loadings greater than 0.5 and belonging to one cluster only were further investigated. Based on these criteria, 409 lipid molecules were identified in 12 clusters (clusters 1, 2, 3, 4, 5, 6, 7, 8, 9, 11, 12, and 16) and analyzed further by stepwise regression. The remaining six clusters (clusters 10, 13, 14, 15, 17, and 18) were excluded from the stepwise regression analysis.

### 3.4. Results of Stepwise Regression Analysis

**Figure 4:**
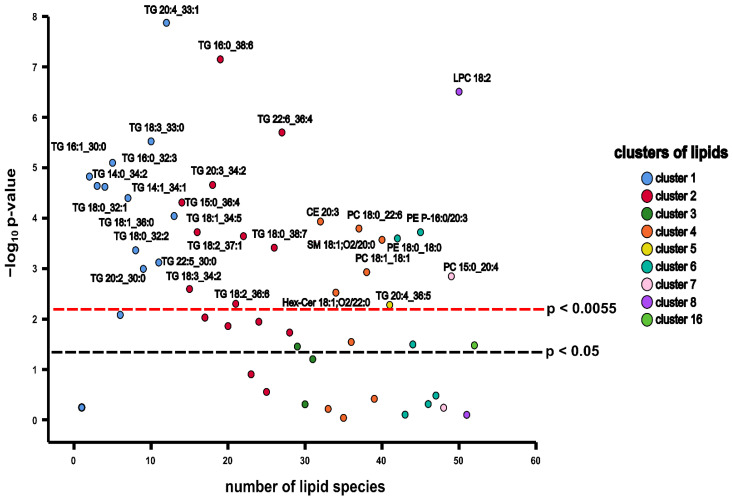
The Manhattan plot of the results from stepwise regression analysis of identified clusters. Each point indicates the negative log_10_ of *p* values obtained in the adjusted regression analysis of individual lipid species. Black dashed line corresponds to the significance level of *p* < 0.05. Red dashed line corresponds to the Bonferroni-adjusted significance level of *p* < 0.0055. Names of only those lipid molecules showing a significant association with BMI after Bonferroni correction are indicated. Abbreviations: CE = cholesterolester, HexCer = hexosylceramide, LPC = lysophosphatidylcholine, PC = phosphatidylcholine, PE = phosphatidylethanolamine, PE P = plasmenylethanolamine, SM = sphingomyelin, TG = triacylglycerol.

Stepwise regression was used to identify lipid species showing a significant association with BMI. From the 409 lipid species (belonging to the 12 clusters), 52 molecules belonging to 9 clusters (clusters 1, 2, 3, 4, 5, 6 7, 8, and 16) were found to show at least nominally significant (*p* < 0.05) association with BMI of which 30 showed (belonging to clusters 1, 2, 4, 5, 6, 7, and 8) a Bonferroni-adjusted significant association (*p* < 0.0055). Figure 4 demonstrates the strength of association (by negative log_10_-transformed p-values) between individual lipid species and BMI. Lipid molecules demonstrating positive relationships with BMI included 27 lipid species belonging to the classes of CEs, PCs, PEs, TGs, and SMs. They are listed in Table 3. Lipid species presenting negative association with BMI comprised three lipid molecules including Hex-Cer 18:1;O2/22:0, LPC 18:2, PC 18:1_18:1 (Table 3.). The relationship was independent of age, sex, and ethnicity.

### 3.5. Results of Selection of Lipid Species Increasing the Strength of Association with Body Mass Index

Compared with the strength of positive association of 27 individual lipid species with BMI, the sum of concentrations of 4 lipid species (PE P-16:0/20:3, TG 20:4_33:1, TG 22:6_36:4, TG 18:3_33:0) increased the level of relationship (*p* = 4.1 × 10^−10^). Compared to the strength of negative association of the three individual lipid species (Hex-Cer 18:1;O2/22:0, LPC 18:2, PC 18:1_18:1) with BMI, the sum of their concentrations also increased the level of relationship (*p* = 8.3 × 10^−8^). Finally, the ratio of the sum of the concentrations of the selected four and three lipid species showing a positive and negative association with BMI, respectively, was calculated. Compared to the concentration of the selected individual lipid molecules, and sum of the levels of lipid species showing positive (*p* = 4.1 × 10^−10^) and those of demonstrating negative (*p* = 8.3 × 10^−8^) association with BMI, the LSR exhibited at least 9 orders of magnitude stronger (*p* = 2.5 × 10^−19^) relationship with BMI. The association was independent of the age, sex, and ethnicity of the individuals.

### 3.6. Estimated Reference Values to Distinguish Normal Weight and Obese Subjects

Reference values were defined for the sum of the concentrations of lipid species showing positive and negative association with BMI as well as LSR. The cut-off value for the sum of the concentration of lipid molecules showing a positive association with BMI was 2.152 µmol/L with 79.2% sensitivity and 77.2% specificity and that for those associating negatively with BMI was 71.426 µmol/L with 66.7% sensitivity and 73.7% specificity. The cut-off value for LSR was 0.029 with 89.6% sensitivity and 87.7% specificity. The estimated reference values and the mean of the sum of concentrations of lipid species showing positive and negative association with BMI as well as LSR in normal weight and obese subject are demonstrated in Table 4.

## 4. Discussion

Lipidomics has greatly advanced by methodological developments allowing more and more detailed mapping of lipid classes and species during the last decade [7,31,32]. However, elucidating the role of tremendous lipid species in obesity-related metabolic changes is still challenging [15]. In this study, we propose a possible approach that can help to process, present, and interpret lipidomic data in profiling lipidomic signature of human obesity.

Analysis of changes at lipid class level do not allow the identification of the key obesity-related lipid species. To investigate the obesity-related metabolic alterations at molecular (species) level, we carried out a more sophisticated lipidomic analysis by introducing a stepwise regression analysis and LSR calculation. In this way, we identified four and three key lipid species showing a strong significant positive (PE P-16:0/20:3, TG 20:4_33:1, TG 22:6_36:4, TG 18:3_33:0) and negative (Hex-Cer 18:1;O2/22:0, LPC 18:2, PC 18:1_18:1) association with BMI, respectively. To demonstrate the combined effect of these individual lipid molecules on the association with BMI, we summed the concentration of lipid species showing positive and negative relationship with BMI. This resulted in a great increase in strength of association. To express the simultaneous effect of the sum of concentrations of lipid species showing positive and negative relationship with BMI in a single parameter, we introduced the lipid species ratio (LSR). Among the parameters concerning the sum of the concentrations of lipid species showing positive and negative association with BMI, and LSR, the LRS demonstrated the highest sensitivity and specificity for obesity. Therefore, it can be used as a sensitive and predictive indicator to monitor obesity-related alterations in human plasma and control the effectiveness of treatment of obesity associated non-communicable diseases.

The concentrations of plasma lipids were measured in an interlaboratory comparison study (ILCS) by Bowden et al. [33]. The levels of lipid species showing a strong positive and negative association with BMI (listed in Table 3) were compared with the concentrations of lipid molecules determined in the ILCS and by Sales et al. when their chemical structures were unequivocally identical with the lipid molecules measured in our study. In this manner, our comparison included four lipid species such as CE 20:3, Hex-Cer 18:1;O2/22:0, LPC 18:2, and SM 18:1;O2/20:0. As shown in Table 5, the average level of CE 20:3 was the same as reported in the ILCS [33]. In addition, the level of LPC 18:2 measured in our study was comparable to that of reported by Sales et al. [34]. However, the concentrations of Hex-Cer 18:1;O2/22:0 and SM 18:1;O2/20:0 were different from those published in the two previous studies. On the other hand, the data presented in Table 5 indicate that there were also differences between the results of the ILCS and that of reported by Sales et al. [34].

The differences between our results and those of the ILCS could be due to the method of plasma sample collection. Aliquots of a standard reference plasma pooled from 100 individuals representing the general population of the United States were analyzed in the ILCS while plasma samples from 136 subjects were examined separately in our investigation. In addition, there could be differences between the dietary habits of the two populations that have been reported to influence the concentrations of plasma lipid species significantly [35]. Comparing our data with the levels of lipid classes published by Sales et al., it can be concluded that the concentrations of Cers, LPCs, and PCs were similar to those of determined in our research as demonstrated in Table 6. However, the levels of CEs, DGs, HexCers, LPEs, PEs, SMs, and TGs were different. The concentration of PEs measured by Bowden et al. was similar to that of determined in our study while the levels of other lipid classes presented in Table 6 were different. One reason of the diverse results could be that there were discrepancies in the number and chemical structure of lipid species belonging to the lipid classes analyzed (Table 6). Therefore, the comparison of the results of different lipidomic studies is challenging and should be considered to perform it at lipid species level. 

Previous studies have demonstrated that the levels of several TGs (TG 48:0, TG 48:1, TG 50:1, TG 50:2, TG 52:1, TG 56:4, TG 56:5) positively correlate with BMI [11,14]. In our study from the 52 lipid molecules showing nominally significant association with BMI, 31 were found to be TGs and 21 of them showed also a significant association with BMI after Bonferroni correction. They were predominantly TG molecules containing more than 50 carbon atoms. In addition, among the sums of the concentrations of lipid species analyzed in our study, the combined levels of three TG molecules (TG 20:4_33:1, TG 22:6_36:4, TG 18:3_33:0) showed an increased positive relationship with BMI. Excessive consumption of Western-style food rich in FAs has been reported to increase the level of FA-derived lipid metabolites in the plasma of overweight and obese persons [36]. FAs not used as an energy source are converted to TGs in the liver and stored in adipose tissue under physiological conditions [37]. During physical activity, TGs are remobilized and transported to muscles where they are decomposed by β-oxidation to serve as energy source in normal weight subjects [38]. Former research has demonstrated that obesity is associated with an enlargement of adipocytes [39]. These hyperthropic cells have limited storage capacity; therefore, TGs are accumulated in non-adipose organs including muscles [40,41]. TGs accumulated in muscles have been reported to inhibit β-oxidation of fatty acids (FAs), which can lead to an increase in the level of toxic lipid species (DGs, Cers) in plasma from overweight and obese individuals [40,42]. However, it has been suggested that not all types of TG molecules can contribute to a reduction in mitochondrial β-oxidation in muscle cells equally [36]. Earlier in vitro and in vivo studies have described that TGs composed of long-chain FAs (FAs incorporating more than 12 carbon atoms) are usually stored in adipocytes and their metabolism in muscles by β-oxidation is considerably slower than that of TGs with FAs containing less than 12 carbon atoms [36,43]. Due to impaired β-oxidation of FAs, muscle cells can become saturated and subsequently TGs comprising long-chain FAs spill over and enter the blood [44]. In this manner, their levels are increased in the plasma of overweight and obese persons [40]. Our results indicate that all of the 21 TGs showing a significant association with BMI included FAs with more than 12 carbon atoms. In addition, the 3 TG molecules (TG 20:4_33:1, TG 22:6_36:4, TG 18:3_33:0) taken into consideration in the calculation of LSR, included even longer FA chains. Therefore, our findings are consistent with the results of previous studies showing a strong association between obesity and high plasma concentrations of TG molecules consisting of long chain FAs.

Candi et al. reported that the concentrations of 10 plasmenylethanolamine species (PE Ps) were increased in adipose tissue of obese individuals [45]. Plasmenylethanolamines have been reported to show an anti-inflammatory effect, thereby protecting cells from oxidative damage [46]. An increase in their levels has been described in obesity and proposed as a compensatory response to obesity-related chronic inflammation [47,48]. Apart from the PE P species described by Candi et al., in our study, the concentration of PE P-16:0/20:3 also showed a strong positive association with BMI. The concentrations of hundreds of lipid species including PE P-16:0/20:3 in human plasma have been described to be dependent on dietary habits [49]. Therefore, the lack of this species in the samples from obese subjects examined by Candy et al. could be due to the differences between the dietary habits of the populations studied. This suggests that the dietary habits should also be considered to clarify the role of PE Ps in obesity-related inflammatory changes.

Wang et al. found a significant decrease in the level of 5 LPC species in obesity such as LPC 18:1, LPC 18:2, LPC 20:0, LPC 20:1, and LPC 20:2 [12]. Our hierarchical statistical approach allowed to select among these LPC species the LPC 18:2 molecule showing the strongest negative association with BMI. Although the exact mechanism underlying a decrease in the level of these LPC species is not known, the role of lecithin cholesterol acyltransferase (LCAT) converting PC to LPC by removing FAs from the PC molecules was suggested [11,12]. LCAT activity has been reported to be decreased in obese subjects resulting in a reduction in plasma LPC levels [12]. It is worth mentioning that in our study there was no significant decrease in the concentration of LPCs in connection with increasing BMI.

In accordance with the result of a previous investigation, the concentration of PC 18:1_18:1 showed also a negative association with BMI in our study [9]. This can be related to an altered activity of Stearoyl Coenzyme A desaturase-1 (SCD-1), the enzyme that converts saturated FAs 18:0 to monounsaturated FAs 18:1, in obesity [9,50]. In addition, monounsaturated FAs have been reported to be oxidized more rapidly than saturated FAs [51,52]. As a result, the concentration of lipid species containing FAs 18:1 such as PC 18:1_18:1 can decrease in the plasma from overweight and obese persons.

In our and a previous study a significant negative association was shown between BMI and the level of HexCers including Hex-Cer 18:1;O2/22:0 [53]. HexCers are produced enzymatically by glucosylceramide synthase from Cers [53,54]. However, HexCers have been suggested to be converted back to Cers in obesity resulting in a decrease in their concentration [53,54]. Our finding supports this assumption however, further research is required to elucidate the role of metabolic balance between Cers and HexCers in obesity-related changes in human plasma lipidome.

In conclusion, the results of our study showed that the trends in the concentrations and representations of lipid classes were significantly associated with BMI categories. The hierarchical statistical approach used in this investigation was suitable for selection of lipid species showing a strong relationship with BMI. The LSR introduced in our study demonstrated the highest sensitivity and specificity for obesity and a stronger association with BMI than individual lipid species alone. However, further studies are required to determine whether the LSR can be used to monitor obesity-related abnormalities in human plasma lipidome.

The strengths and limitations of this study should be considered. One strength of our investigation is that large number of lipid species belonging to 11 lipid classes were measured simultaneously allowing a comprehensive analysis of obesity-related alterations in human plasma lipidome. Second, we used a hierarchical statistical approach to select the lipid species strongly associated with BMI. Third, the results of stepwise regression analyses were independent of the sex, age, and ethnicity. Forth, we introduced a new LSR showing a stronger association with BMI than individual lipid species alone. Our research also has several limitations. First, the number of subjects involved in our study was relatively low. Second, this was a cross-sectional study, which does not allow to answer the question whether the changes in lipidomic profile are the cause or consequence of obesity. Third, we investigated the association between representation/concentration of lipid species and BMI, which is only one of several parameters characterising nutritional status. Fourth, we used electrospray ionization, which is a “soft” ionization technique, in which the fragmentation of analytes have been reported to be minimal [55], however, theoretically it cannot be excluded [56]. Concerning the fact that in our present study group comparisons were made, if a small portion of fatty acids comes from in-source fragmentation it results in a systematic error which applies to all groups.

## Figures and Tables

**Table 1:** Demographic and anthropometric parameters of the study population.

	BMI < 25 kg/m^2^			BMI 25–29.9 kg/m^2^			BMI ≥ 30 kg/m^2^			
	*n*	Rep (%)	Mean Age ± SD (years)	*n*	Rep (%)	Mean Age ± SD (years)	*n*	Rep (%)	Mean Age ± SD (years)	Total *n*
total	57	41.91	41.61 ± 11.23	31	22.79	45.03 ± 10.75	48	35.29	49.06 ± 9.74	136
male	16	28.07	45.94 ± 12.57	12	38.71	43.00 ± 12.02	14	29.17	49.36 ± 8.18	42
female	41	71.93	39.93 ± 10.34	19	61.29	46.32 ± 9.99	34	70.83	48.94 ± 10.43	94
Hungarian general	31	54.39	42.26 ± 11.45	16	51.61	46.00 ± 10.61	24	50.00	51.67 ±8.38	71
Hungarian Roma	26	45.61	40.85 ± 11.13	15	48.39	44.00 ± 11.17	24	50.00	46.46 ± 10.47	65

Abbreviations: BMI = body mass index, *n* = number of subjects, rep = representation.

**Table 2:** Clinical characteristics of the study population.

	Reference Value *	BMI < 25 kg/m^2^	BMI 25–29.9 kg/m^2^	BMI ≥ 30 kg/m^2^	*p* for Trend
		Mean ± SD	Mean ± SD	Mean ± SD	
TC (mmol/L)	<5.2 mmol/L in both sexes [27]	4.70 ± 0.95	5.06 ± 0.87	5.39 ± 0.92	<0.001
TG (mmol/L)	<1.7 mmol/L in both sexes [28]	1.02 ± 0.50	1.37 ± 0.69	1.76 ± 0.88	<0.001
HDL-C (mmol/L)	<1.03 mmol/L in male and <1.29 mmol/L in female [28]	1.47 ± 0.32	1.27 ± 0.33	1.30 ± 0.36	<0.010
TG/HDL ratio	<1 for both sexes [29]	0.77 ± 0.50	1.22 ± 0.84	1.54 ± 1.06	<0.001
LDL-C (mmol/L)	<3.3 mmol/L in both sexes [30]	2.95 ± 0.86	3.34 ± 0.88	3.47 ± 0.96	<0.010
ApoAI (g/L)	<1.2 g/L in male and < 1.4 g/L in female [30]	1.54 ± 0.23	1.44 ± 0.26	1.55 ± 0.29	>0.050
ApoB (g/L)	<1.3 g/L in both sexes [30]	0.96 ± 0.25	1.10 ± 0.24	1.18 ± 0.23	<0.001

* The reference values were obtained from previous studies (see references 27–30). Abbreviations: BMI = body mass index, TC = total cholesterol, TG = triacylglycerol, HDL-C = high density lipoprotein-cholesterol, LDL-C = low density lipoprotein-cholesterol, ApoAI = apolipoprotein AI, ApoB = apolipoprotein B.

**Table 3:** The lipid species used in the stepwise calculation of lipid species ratio and their effect on the association with BMI.

Direction of Association with BMI	No. of Step	Lipid Species	Negative log_10_-Transformed *p* Values	Increase in the Strength of Association with BMI
**positive**	**1**	**TG 20:4_33:1**	**7.876**	**r.l.m.**
	2	TG 16:0_38:6	7.428	no
	**3**	**TG 22:6_36:4**	**9.056**	**yes**
	**4**	**TG 18:3_33:0**	**9.175**	**yes**
	5	TG 16:0_32:3	6.580	no
	6	TG 16:1_30:0	6.000	no
	7	TG 20:3_34:2	5.398	no
	8	TG 14:0_34:2	4.921	no
	9	TG 14:1_34:1	6.034	no
	10	TG 18:0_32:1	4.921	no
	11	TG 15:0_36:4	6.854	no
	12	TG 18:1_36:0	4.678	no
	13	CE 20:3	4.260	no
	14	PC 18:0_22:6	4.337	no
	15	TG 18:1_34:5	8.276	no
	**16**	**PE P-16:0/20:3**	**9.387**	**yes**
	17	TG 18:2_37:1	8.824	no
	18	PE 18:0_18:0	9.337	no
	19	SM 18:1;O2/20:0	4.469	no
	20	TG 18:0_38:7	9.214	no
	21	TG 18:0_32:2	5.699	no
	22	TG 22:5_30:0	8.377	no
	23	TG 20:2_30:0	9.125	no
	24	PC 15:0_20:4	8.509	no
	25	TG 18:3_38:2	3.292	no
	26	TG 20:4_36:5	8.602	no
	27	TG 18:2_36:6	8.409	no
**negative**	**1**	**LPC 18:2**	**6.509**	**r.l.m.**
	**2**	**PC 18:1_18:1**	**7.060**	**yes**
	**3**	**Hex-Cer 18:1;O2/22:0**	**7.079**	**yes**

*p* values were obtained when considering the association between the sum of concentration of lipid species and BMI. The association was evaluated under adjusted (by sex, age, and ethnicity) regression models. A higher value of the negative log_10_-transformed p values indicates a stronger association between the sum of lipid species concentration and BMI. Red and green rows show lipid species showing a positive or negative association with body mass index (BMI) with a strength of association increasing effect, respectively. Abbreviations: CE = cholesterolester, HexCer = hexosylceramide, LPC = lysophosphatidylcholine, PC = phosphatidylcholine, PE = phosphatidylethanolamine, PE P = plasmenylethanolamine, SM = sphingomyelin, TG = triacylglycerol, r.l.m. = reference lipid molecule.

**Table 4:** The estimated reference values and the mean of the sum of concentrations of lipid species showing positive and negative association with body mass index and lipid species ratio.

	Reference Value	BMI < 25 kg/m^2^	BMI ≥ 30 kg/m^2^	*p* Value
		Mean ± SD	Mean ± SD	
Sum of the concentration of lipid molecules showing positive association with BMI	<2.15 µmol/L	1.54 + 0.76	2.73 + 0.73	<0.001
Sum of the concentration of lipid molecules showing negative association with BMI	≥71.43 µmol/L	83.92 + 22.33	63.38 + 21.55	<0.001
Lipid species ratio	<0.03	0.02 + 0.01	0.05 + 0.03	<0.001

**Table 5:** Comparison of the concentrations of lipid species measured in different studies.

Lipid Species	This Study Mean ± SD * (µmol/L)	Bowden et al. ** Mean ± SD (µmol/L)	Sales et al. ** Mean ± SD *** (µmol/L)
CE 20:3	34.46 ± 10.27	35 ± 12	17.51 ± 5.97
Hex-Cer 18:1;O2/22:0	20.81 ± 4.86	2.4 ± 0.68	3.20 ± 0.73
LPC 18:2	60.52 ± 20.12	22 ± 2.9	54.26 ± 13.17
SM 18:1;O2_20:0	0.87 ± 0.26	11 ± 3.1	11.66 ± 2.09

* Data on the levels of lipid species in the plasma of normal weight subjects were averaged. ** Data on the concentrations of lipid species were collected from the publication by Bowden et al. and Sales et al. [34]. *** Data on the levels of lipid species in the plasma of males and non-contraceptive user females were averaged. Mean values ± standard deviations (SD) are demonstrated.

**Table 6:** Comparison of the concentrations of lipid classes determined by Bowden et al., Sales et al., and in this study.

Lipid Class	This Study		Bowden et al. *		Sales et al. *	
	Concentration Mean ± SD (µmol/L)	Number of Lipid Species Measured within the Lipid Class	Concentration Mean ± SD (µmol/L)	Number of Lipid Species Measured within the Lipid Class	Concentration Mean ± SD ** (µmol/L)	Number of Lipid Species Measured within the Lipid Class
CEs	2070.25 ± 508.82	14	2981 ± 450	16	3473.58 ± 484.08	15
Cers	7.31 ± 1.95	5	- ***	-	5.33 ± 1.28	8
DGs	21.63 ± 11.26	12	53 ± 7	23	40.80 ± 13.59	12
HexCers	1.72 ± 0.58	2	- ***	-	17.30 ± 3.38	9
LPCs	291.2 ± 82.82	9	153 ± 12	12	276.92 ± 37.11	13
LPEs	4.54 ± 1.48	4	7 ± 1	7	13.99 ± 3.15	7
PCs	1503.76 ± 339.08	40	1074 ± 68	31	1278.37 ± 202.95	29
PEs	61.95 ± 17.69	28	70 ± 4	31	23.43 ± 10.43	10
SMs	77.12 ± 15.14	4	334 ± 22	35	318.72 ± 45.62	26
TGs	2756.56 ± 1439.95	424	491 ± 46	18	628.79 ± 225.66	48

* Data on the concentrations of lipid classes were collected from the publication by Bowden et al. and Sales et al. [34]. ** Data on the levels of lipid classes in the plasma of males and non-contraceptive user females were averaged. Mean values ± standard deviations (SD) are demonstrated. *** The sum of the concentration of Cer and HexCer was defined therefore, their levels can not be shown separately. Abbreviations: CEs = cholesterolesters, Cers = ceramides, DGs = diacylglycerols, HexCers = hexosylceramides, LPCs = lysophosphatidylcholines, LPEs = lysophosphatidylethanolamines, PCs = phosphatidylcholines, Pes = phosphatidylethanolamines, SMs = sphingomyelins, TGs = triacylglycerols.

## Data Availability

The data underlying this article cannot be shared publicly due to the privacy of the participants of the project (GINOP-2.3.2-15-2016-00005) and legal reasons (study participants did not sign informed consent to make their data publicly available). The data will be available upon request to interested qualified researchers. Data requests should be sent to the corresponding author.
